# A Fully Self-Healing Piezoelectric Nanogenerator for Self-Powered Pressure Sensing Electronic Skin

**DOI:** 10.34133/2021/9793458

**Published:** 2021-04-14

**Authors:** Maosen Yang, Jinmei Liu, Dong Liu, Jingyi Jiao, Nuanyang Cui, Shuhai Liu, Qi Xu, Long Gu, Yong Qin

**Affiliations:** ^1^School of Advanced Materials and Nanotechnology, Xidian University, Xi'an 710071, China; ^2^Institute of Nanoscience and Nanotechnology, Lanzhou University, Gansu 730000, China

## Abstract

As an important way of converting mechanical energy into electric energy, a piezoelectric nanogenerator (PENG) has been widely applied in energy harvesting as well as self-powered sensors in recent years. However, its robustness and durability are still severely challenged by frequent and inevitable mechanical impacts in real application environments. Herein, a fully self-healing PENG (FS-PENG) as a self-powered pressure sensing electronic skin is reported. The self-healing piezoelectric composite and self-healing Ag NW electrode fabricated through mixing piezoelectric PZT particles and conductive Ag NWs into self-healing polydimethylsiloxane (H-PDMS) are assembled into the sandwich structure FS-PENG. The FS-PENG could not only effectively convert external stimulation into electrical signals with a linear response to the pressure but also retain the excellent self-healing and stable sensing property after multiple cycles of cutting and self-healing process. Moreover, a self-healing pressure sensor array composed of 9 FS-PENGs was attached on the back of the human hand to mimic the human skin, and accurate monitoring of the spatial position distribution and magnitude of the pressure was successfully realized.

## 1. Introduction

With the wide application of micro/nanoelectronic devices, the increasing demand for batteries causes serious environment issues. Self-powered devices that can harvest energy from ambient environment have been proved to be an effective power supply without environmental pollution [1–3]. Generally speaking, the nanogenerator (NG) is capable of converting mechanical energy into electrical energy based on the piezoelectric effect (PENG) [4–6] or triboelectric effect (TENG) [7–9]. With the merits of high conversion efficiency, low cost, diversified designability, and easy fabrication, NGs have shown immense potential in various applications as a green sustainable power source or a battery-free sensor such as portable/wearable devices [10, 11], artificial skins [12, 13], and sensor networks [14]. However, considering that polymers are the most commonly used matrix materials in PENGs and friction layers and substrate materials in TENGs [15], unpredicted damage could be inevitably induced by external mechanical action such as violent interface friction, frequent deformation, and accidental cut and scratch, resulting in the degradation of output performance or even failing to work. Inspired by self-healing property of biological skin, the development of advanced self-healing NGs is an effective strategy to handle this problem [16–22].

In recent years, massive efforts have been made to design and fabricate self-healing NGs. For example, Parida's group adopted the self-healing ionic conductors as electrodes of TENG to improve the output performance and the stability of the devices and successfully applied them in human skin tactile sensing [17]. Sun's group combined the conductive silver nanowires (Ag NWs) and self-healing polydimethylsiloxane to fabricate a self-healing and scalable TENG. The TENG was utilized as a flexible electronic skin that could reach 100% of the initial output value after cutting and repairing [18]. Moreover, as an electronic device physically contacting with human skin, the synergistic interaction between the device and the human skin is particularly important. Toward this, Dai's group designed a self-healing and scalable TENG by introducing the reversible imine and tetrahedral hydrogen bonds (UPy) to the polymer network. Making use of the spontaneous infrared radiation of human skin to promote the self-healing of the friction layers, a wearable motion monitoring sensor for human-computer interaction was successfully realized [22]. However, TENGs are very sensitive to the change of working environment such as humidity [23, 24], temperature [25, 26], and dust particles [27, 28] so that its output performance is unstable which is not conductive to the sensing system. Considering PENG's stability is much stronger than TENG [29–33], if the self-healing strategy could be introduced to design PENG, it will be very helpful to the development and application of NGs, especially in self-powered sensing system.

In this work, a fully self-healing PENG (FS-PENG) with sandwich structure was developed. It can effectively convert external stimulation into electrical signals exhibiting a linear response to the pressure, retain the excellent self-healing and stable sensing property after multiple cycles of cutting and self-healing process, and be utilized as a self-powered pressure sensing electronic skin.

## 2. Result and Discussions

### 2.1. Design and Fabrication of FS-PENG

**Figure 1:**
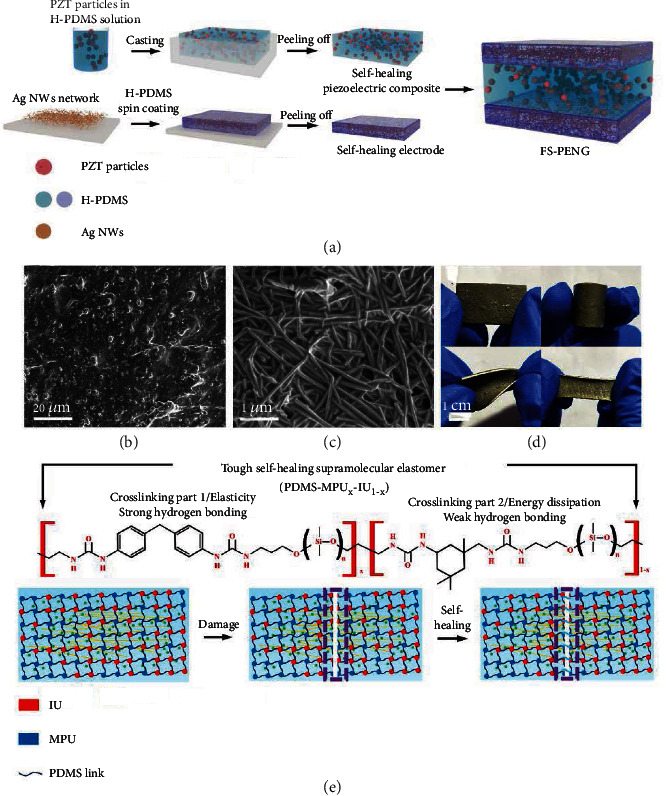
Fabrication and characterization of FS-PENG. (a) Schematics of the FS-PENG's fabrication process. (b, c) SEM images of self-healing piezoelectric composite and self-healing Ag NW electrode. (d) Optical images of FS-PENG in initial, bending, twisting, and stretching states. (e) The chemical structure formula and self-healing mechanism of H-PDMS.

In the fabrication process of FS-PENG, 4,4′-methylene bis phenyl urea (MPU) and isofordone biuret (IU) were firstly added into double 3-amino-propyl terminated polydimethylsiloxane (NH_2_-PDMS-NH_2_, *M*_n_ = 5000) and mixed uniformly to form the precursor solution of H-PDMS. Subsequently, PZT particles and conductive Ag NW networks were incorporated with H-PDMS solution, respectively, to get the self-healing piezoelectric composite and Ag NW electrode. The SEM image and X-ray diffraction graph of PZT particles were shown in Figure S1. The detailed fabrication process of H-PDMS and the Ag NW conductive network were described in Materials and Methods. Finally, a FS-PENG with a sandwich structure could be simply assembled by the piezoelectric composite and Ag NW electrodes due to abundant dynamic hydrogen bonds in H-PDMS [34], as shown in Figure 1(a). Surface morphology of the self-healing PZT composite and Ag NW electrode was characterized by SEM (Figures 1(b) and 1(c)). It could be found that PZT particles were uniformly dispersed in H-PDMS (Figure 1(b)). In addition, we also found that numerous Ag NWs were closely connected to each other constructing a network structure that is embedded in H-PDMS (Figure 1(c)). The network structure provided an essential foundation for reconnection between Ag NWs in the self-healing process of H-PDMS. Optical images of the FS-PENG with a sandwich structure were shown in Figure 1(d). The thin gray layers on the top and bottom were two self-healing Ag NW electrodes, and the thick white layer in the middle was self-healing PZT composite. Benefitting from the superior mechanical property of H-PDMS, the FS-PENG could be bended, twisted, and stretched without damage, as shown in Figure 1(d), which exhibits excellent flexibility. The chemical structure formula and self-healing mechanism of H-PDMS were schematically shown in Figure 1(e). MPU and IU molecules could incorporate with NH_2_-PDMS-NH_2_ forming elasticity strong hydrogen bonding (Crosslinking part 1) and weak hydrogen bonding (Crosslinking part 2), respectively, through polycondensation reaction. These interactions lead to the formation of the cross-linked polymer network of H-PDMS, which provides the elastic mechanism and energy dissipation mechanism, respectively, for the self-healing process of H-PDMS [34]. Specifically, strong hydrogen bonding that resulted from MPU provides high tensile property which is important for flexible electronics, while weak hydrogen bonding resulted by IU contributes greatly to the self-healing property. Moreover, IU also acts as an important role in reducing glass transition temperature (*T*_g_) of H-PDMS by preventing agglomeration of a large number of MPU. Therefore, because of abundant dynamic hydrogen bonds in the elastomer and the low *T*_g_ (lower than room temperature) of the H-PDMS backbone, FS-PENG could exhibit excellent self-healing property in absence of external stimulus at room temperature.

### 2.2. Self-Healing Property of FS-PENG

**Figure 2:**
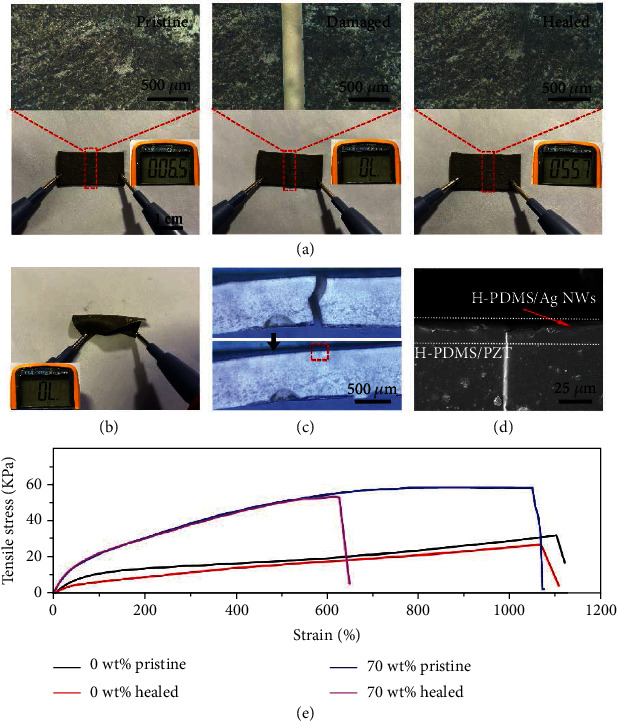
Characterization of the FS-PENG's self-healing property. (a) Top view images of FS-PENG and the resistance variation of top Ag NW electrode before and after self-healing. (b) Conducting state between the top and bottom Ag NW electrodes after self-healing. (c, d) Cross-section images of the FS-PENG. Optical images of the FS-PENG (c) before and after self-healing, and (d) SEM image of the healed FS-PENG. (e) The stress and strain curves of FS-PENG with PZT mass ratio of 0 wt% and 70 wt% before and after self-healing.

To verify the self-healing property of the FS-PENG, a device with size of 2.8 cm × 1.5 cm was cut into two separate parts, as shown in Figure 2(a). From left to right, the optical images were FS-PENG in the state of before cutting, after cutting, and after healing. It could be seen that the two parts recombined to be a whole one when putting them together without any external stimulus at room temperature. The corresponding resistances of the Ag NW electrode in the above three states were also measured by multimeter. The pristine electrode showed good conductivity with resistance value of 6.5 *Ω*, and then, the value changed to be overload (ranges of multimeter was 20 M*Ω*) which meant that the electrodes on the divided two parts were disconnected. After the self-healing process, the resistance recovered to about 55.7 *Ω* which demonstrated that the conductivity of the electrode was almost completely healed on account of the reconnection of the Ag NW network structure. The same operation was conducted on the bottom electrode, and its conductivity was also recovered. Normally, the two electrodes of PENG should not be short circuit so that the potential difference between the two electrodes could be created by external stimulus. To verify the conducting state between the top and bottom Ag NW electrodes after the self-healing process, resistance between the two electrodes was measured and shown in Figure 1(b). The resistance value showed overload (ranges of multimeter was 20 M*Ω*) demonstrating that the two electrodes were not in cross-contact after the healing process. The cross-section images of the FS-PENG after healing were characterized and shown in Figures 1(c) and 1(d). No crevices could be found between the interface of Ag NW electrodes and PZT composite (70 wt%), meanwhile, and the incision was perfectly healed, which demonstrated the excellent self-healing property of FS-PENG. Moreover, the tensile test was conducted to FS-PENGs without PZT and with 70 wt% PZT at a uniaxial tensile speed of 10 mm/min, as shown in Figure 2(e). For the FS-PENG without PZT, the ultimate breaking strain was 1100% at stress of 32 kPa (black curve). Subsequently, it was cut into two parts and then healed at room temperature for 12 hours, and the mechanical property of this device recovered almost to its initial state (red curve). The same test was adopted to FS-PENG with PZT (70 wt%); the stress-strain curves before it was damaged (blue curve) and after healing (purple curve) were recorded. It could be found that the modulus of FS-PENG was increased while the self-healing property was weakened due to the presence of PZT particles which limited the free movement of dynamic hydrogen bonds. Although the mechanical property of FS-PENG (70 wt% PZT) after self-healing was weakened, it was enough to meet the design and application demand of flexible electronic devices with self-healing property.

### 2.3. Working Mechanism and Output Performance of FS-PENG

**Figure 3:**
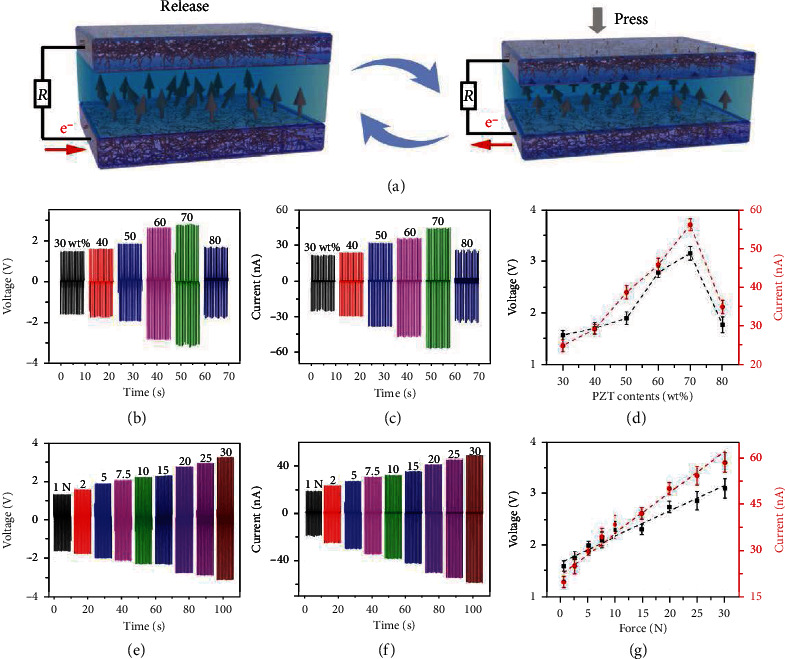
Working mechanism and output performance of FS-PENG. (a) Schematic diagram of PENG's working mechanism. (b–d) Effect of PZT contents on the output voltage and current of FS-PENG. (e–g) Effect of external pressure on the output voltage and current of FS-PENG with 70 wt% PZT.

The working mechanism of FS-PENG was schematically shown in Figure 3(a). When periodic deformation was applied on the FS-PENG by external stimulus, electrical dipole moments in PZT particles would induce a periodically changing potential difference between the two electrodes. As a result, the free electrons in the external circuit could be driven to flow back and forth to generate AC electric output signals. Because the piezoelectric effect of FS-PENG was contributed by PZT particles, the content of PZT particles greatly influences the output of FS-PENG. With the driven force of 20 N, the electric output of FS-PENG with size of 1 cm × 1 cm was characterized and shown in Figures 3(b)–3(d). It could be found that the output voltage and current increased with the content of PZT particles increasing from 30 wt% to 70 wt%, and then decreased when PZT content further increased to 80 wt%. The reason for this tendency could be explained as follows: PENG could be considered a capacitor in which the electric charge was generated by the piezoelectric effect [35]. With the filler content of PZT particles increased in the composite, both of its piezoelectric coefficient and dielectric constant are improved. Higher piezoelectric coefficient improves the charge quantities which contributes to a higher output of FS-PENG. Compared with the piezoelectric coefficient, higher dielectric constant will lower the capacitance causing decreased output of the FS-PENG. In addition, compositional and structural inhomogeneity when mixing too many PZT particles into the composite could also easily lead to breakdown [36], resulting in a decreased piezoelectric coefficient of the piezoelectric composite. Based upon the interaction of the above factors, the output performance of FS-PENG exhibited a tendency of first increase and then decrease with the increasing of PZT content and generated the maximum output voltage and current of 3.2 V and 56.1 nA at the PZT content of 70 wt%. To demonstrate that the output signals were contributed by the piezoelectric effect rather than the triboelectric effect, we measured the outputs of FS-PENGs with 0 wt% and 70 wt% PZT particles before and after polarization (Figure S2). As shown in Figure S2a and b, the output current and voltage of FS-PENG with 0 wt% PZT particles had no obvious difference before and after polarization, and both of their values were much smaller than that of FS-PENGs with PZT particles in Figures 3(b) and 3(c). As for the FS-PENG with 70 wt% PZT particles, its output before polarization was in the same level with that of the one with 0 wt% PZT particles, but the output could be substantially increased after it was polarized (Figure S2c and d). These results could clearly show that the output of our FS-PENGs resulted from the ordered aligned dipole moments in the PZT particles which was same with that we discussed in Figure 3(a). Moreover, FS-PENG was very sensitive to the change of driven pressure. Due to the output of PENG being positively correlated to external pressure, the output current and voltage of FS-PENG gradually increased with the pressure increasing from 1 N to 30 N, as shown in Figures 3(e) and 3(f). The corresponding peak to peak values of output voltage and current were presented in Figure 3(g), and a good linearity relationship could be observed between the output and external pressure. This characteristic was very profitable to the application of PENG in the aspect of flexible pressure sensing.

**Figure 4:**
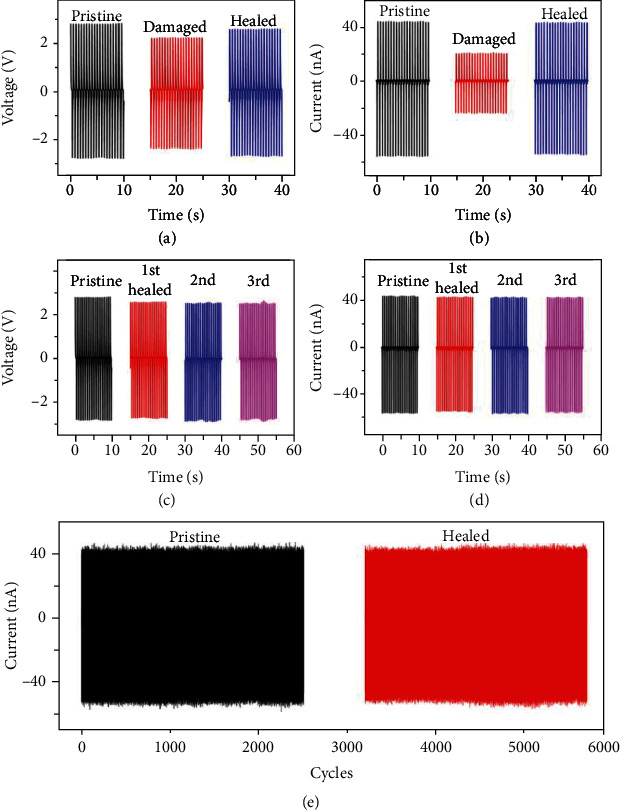
Influence of self-healing process on the output voltage and current of FS-PENG. (a, b) The output voltage and current FS-PENG in pristine (black), damaged (red), and healed (blue) states. (c, d) The output voltage and current after multi cycles of cutting and healing. (e) Working stability test of FS-PENG before cutting and after self-healing.

To investigate the change of FS-PENG's output performance before and after self-healing, the output voltage and current of FS-PENG in pristine (black), damaged (red), and healed (blue) states were recorded and shown in Figures 4(a) and 4(b). When the FS-PENG was damaged, both of the output voltage and current were dropped down. In contrast, the output current was decreased more than voltage. It was mainly due to that the output voltage depended more on the device's thickness, while the output current depended more on the device's active area. When the FS-PENG was cut into two separate parts, only the active area was dramatically decreased for the device. The output of FS-PENG could almost recover to the initial value after self-healing. As the electronic devices might be damaged frequently, the repeated self-healing property of FS-PENG was very important for its application in our real life. As shown in Figures 4(c) and 4(d), the output of FS-PENG after three cycles of cutting and self-healing processes showed no obvious difference compared to the pristine one (Figures 4(c) and 4(d)). Besides, FS-PENG's output also exhibited excellent stability before and after self-healing (Figure 4(e)). These results demonstrated that our FS-PENG not only had stable output performance but also possessed superior and sustained self-healing property.

### 2.4. Application of FS-PENGs as a Pressure Monitoring Electronic Skin

**Figure 5:**
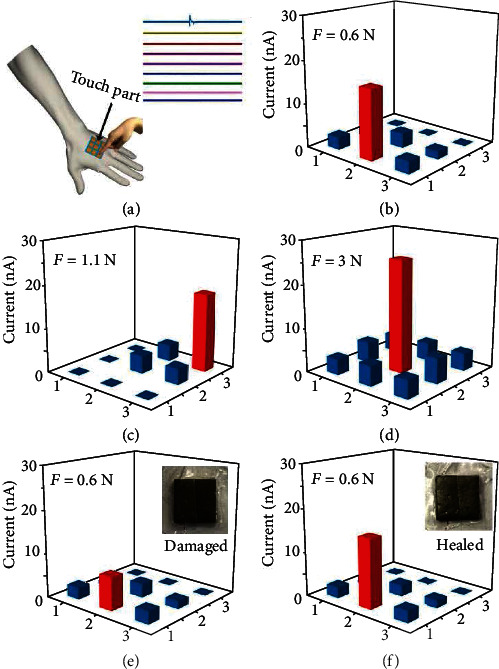
Application of FS-PENGs as a self-powered and self-healing pressure monitoring electronic skin. (a) Schematic of the pressure monitoring electronic skin on the back of human hand. (b–d) Pressure and position detecting property of the pressure monitoring electronic skin. (e, f) Self-healing property of the pressure monitoring electronic skin.

The sensitive response to external pressure and excellent self-healing property of FS-PENG make it be capable of being applied as a pressure monitoring electronic skin. To simultaneously realize the pressure and position detecting, 9 FS-PENGs with size of 0.5 cm × 0.5 cm were combined together as a self-powered sensing arrays and then attached on the back of a human hand acting as a bionic electronic skin, which was shown in Figure 5(a). When the external stimulus was applied on the electronic skin, the measure system recorded 9 parallel output signals generated by each FS-PENG at the same time. Considering that only the FS-PENGs stimulated for force could generate electric output signals, therefore, the spatial position distribution and magnitude of the pressure could be identified according to the electric output mapping of the electronic skin, as shown in Figures 5(b)–5(d). As one main sense of the human body in contact with the outside world, the skin was immensely impacted by the surroundings often causing contused or cut. To the traditional sensors and electrodes, these natural damages were easy to cause irreversible damage resulting in failure of their functionality. Our FS-PENG was capable of perfectly solving this problem. As shown in Figure 5(e), when one FS-PENG unit was cut into two parts, its output current was significantly weakened which could not provide a correct response to the external stimulus. Through the self-healing process, the two parts reintegrated as a whole one presenting superior self-healing property (Figure 5(f)). Meanwhile, the induced response signal also returned to the initial value which could be seen in Figure 5(f). This function demonstrated the great potential of our FS-PENG in the design and implementation of the self-powered and self-healing electronic skin.

## 3. Conclusion

In this work, we developed a FS-PENG and demonstrated its application as a self-powered electronic skin for pressure detection. By adjusting the mass ratio of PZT particles in piezoelectric composites, the piezoelectric property of FS-PENG was optimized. At the PZT content of 70 wt%, the maximum output voltage and current reached about 3.2 V and 56.1 nA, respectively. The linear response to driven pressure indicated good sensitivity of FS-PENG to the external pressure change. The results of the tensile experiment and repeated cutting testing showed excellent self-healing and stable sensing properties of FS-PENG. These abilities above were successfully used in constructing a self-healing pressure sensor array to mimic the human skin, which could accurately identify the spatial position distribution and magnitude of the external stimulus. This work provides a feasible way for the design and implementation of a self-powered and self-healing pressure sensor for the development of the electronic skin.

## 4. Materials and Methods

### 4.1. Preparation of H-PDMS Solution

The typical steps for preparing healable PDMS-MPU_*x*_-IU_1−__*x*_ polymer solution were as follows: firstly, mixing 0.20 g IU and 0.27 g MPU into 8 ml of dichloromethane (CH_2_Cl_2_), and stirring for one hour to obtain a milky white turbid solution. Secondly, 10 g NH_2_-PDMS-NH_2_ and 0.3 g triethylamine were added into 36 ml CH_2_Cl_2_, and then stirred vigorously for 1 hour in ice bath to obtain a uniformly dispersed solution. Thirdly, the above two solutions were mixed together with vigorously stirring in ice bath conditions for 1 hour and then taken the mixed solution out and continue stirring at room temperature for 3-4 days. Finally, the H-PDMS solution was obtained.

### 4.2. Preparation of Ag NW Self-Healing Electrode

The preparation of the self-healing Ag NW electrode included two steps. Firstly, Ag NW alcohol dispersion (12 mg/ml, Nanjing XFNANO Material Technology Co. Ltd.) was sprayed on a PTFE substrate forming the Ag NW network and then annealed at 100°C for 2 hours to improve its density and conductivity. Secondly, the prepared H-PDMS solution was spin-coated on the annealed Ag NW network and cured at 60°C for 2 hours. Finally, the Ag NW self-healing electrode could be peeled off from the PTFE substrate.

### 4.3. Preparation of Self-Healing Piezoelectric Composite Film

Surface-modified PZT particles (30, 40, 50, 60, 70, and 80 wt%) were uniformly mixed in the H-PDMS polymer solution by ultrasonic and stirring, and then, the solution was poured into a 6 cm × 6 cm polytetrafluoroethylene (PTFE) mold. After volatilization of the solvent at room temperature, the composite film could be peeled off from the mold forming a free-standing piezoelectric composite layer.

### 4.4. Fabrication of FS-PENG

The 10 mm × 10 mm × 0.012 mm of Ag NW electrodes (top and bottom layers) and 10 mm × 10 mm × 0.6 mm of the piezoelectric composite (middle layer) were stacked in sequence as shown in Figure 1(a) forming a sandwich structure. Through the self-healing process which resulted from dynamic hydrogen bonds, the three layers could be assembled as a FS-PENG without any crevices. Finally, the FS-PENG was polarized by a direct electric field of 4 kV/mm.

### 4.5. Measurement and Characterization

The mechanical tensile test was carried out with the ESM301/Mark-10 system. The size of the samples was 10 mm × 5 mm × 0.6 mm, and the tensile speed was fixed at 10 mm/min. In the self-healing test, the sample was completely cut into two parts by a commercial razor blade and then healed at room temperature. In the measurement of FS-PENG's output, a linear motor (Linmot E1100) was used to provide the mechanical stimulation. Low-noise voltage and current preamplifier (SR 560 and 570, Stanford) were used to record the output voltage and current.

## Data Availability

All data needed to evaluate the conclusions in the paper are presented in the paper and/or the Supplementary Materials. Additional data related to this paper may be requested from the authors.

## Supplementary Materials

Supplementary Materials Figure S1: SEM image and XRD graph of PZT particles. Figure S2: the output performance of FS-PENG with 0 wt% and 70 wt% PZT particles before and after polarization.
