# Supplementary material for: A Fully Self-Healing Piezoelectric Nanogenerator for Self-Powered Pressure Sensing Electronic Skin
Source: Research (Wash D C). 2021 Apr 14;2021:9793458. doi: 10.34133/2021/9793458 (PMC8063864; doi:10.34133/2021/9793458)
Supplement: Supplementary Materials — Figure S1: SEM image and XRD graph of PZT particles. Figure S2: the output performance of FS-PENG with 0 wt% and 70 wt% PZT particles before and after polarization. [file 9793458.f1.docx]

**A Fully Self-Healing Piezoelectric Nanogenerator for Self-Powered Pressure Sensing Electronic Skin**

Maosen Yang,^1^ Jinmei Liu,^1^ Dong Liu, ^1^ Jingyi Jiao,^1^ Nuanyang Cui,^1^ Shuhai Liu,^2^ Qi Xu,^1^ Long Gu,^1,*^ Yong Qin^2,*^

*^1^School of Advanced Materials and Nanotechnology, Xidian University, Xi’an 710071, China.*

*^2^Institute of Nanoscience and Nanotechnology, Lanzhou University, Gansu 730000, China.*

^*^Corresponding author Email: lgu@xidian.edu.cn, qinyong@lzu.edu.cn


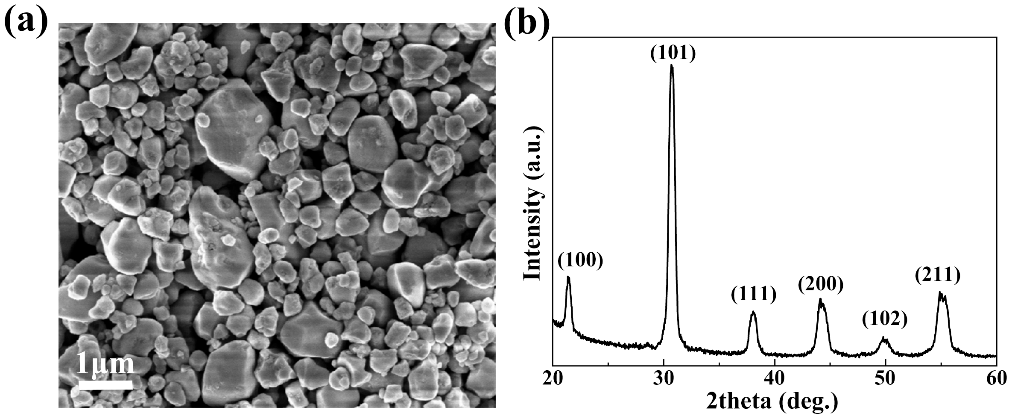


**Figure S1**. **Characterization of PZT particles.** (a) SEM image of PZT particles. (b) X-ray diffraction graph of PZT particles.


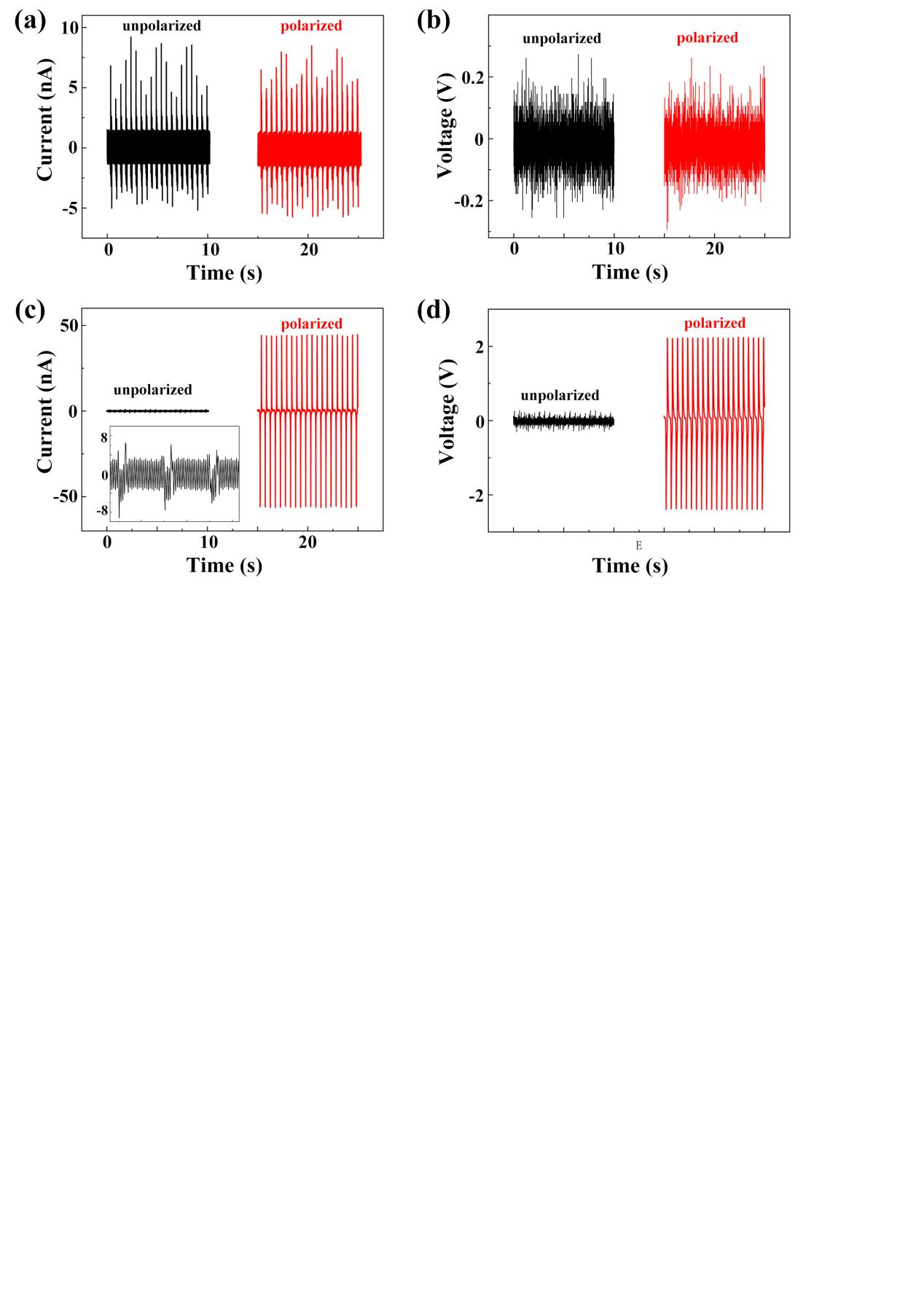


**Figure S2**. **The output performance of FS-PENG with 0 wt% and 70 wt% PZT particles before and after polarization.** (a, b) The output current and voltage of FS-PENG with 0 wt% PZT particles before and after polarization. (c, d) The output current and voltage of FS-PENG with 70 wt% PZT particles before and after polarization.
